# Being Female and in a Romantic Relationship Enhances the Association between Satisfaction with Love Life and Capacity to Love

**DOI:** 10.3390/ijerph20237108

**Published:** 2023-11-24

**Authors:** Ângela Leite, Ana Costa, Beatriz Ribeiro, Carolina Fonseca, Inês Ribeiro, Joana Mesquita, Sara Ribeiro

**Affiliations:** 1 Centre for Philosophical and Humanistic Studies, Faculty of Philosophy and Social Sciences, Universidade Católica Portuguesa, Rua Camões, 4710-362 Braga, Portugal; 2 Department of Education and Psychology, School of Human and Social Sciences, University of Trás-os-Montes and Alto Douro, Quinta de Prados–Folhadela, 5000-801 Vila Real, Portugal; al68281@alunos.utad.pt (A.C.); beatrizborgesribeiro@hotmail.com (B.R.); carolina_gfonseca@hotmail.com (C.F.); diasinesribeiro@gmail.com (I.R.); joanaines690@gmail.com (J.M.); saracriscribeiro@gmail.com (S.R.)

**Keywords:** capacity to love, gender differences, romantic relationship, satisfaction with love life

## Abstract

This study aims to evaluate if the relation between love life satisfaction, assessed by the satisfaction with love life scale (SWLLS), and capacity to love, assessed by the capacity to love inventory (CTL-I), is moderated by gender and by being or not in a romantic relationship, in a Portuguese sample. To this end, the adaptation and validation of CTL-I for this population were carried out through an exploratory factorial analysis (EFA) followed by a Robust Maximum Likelihood (MLR) confirmatory factorial analysis (CFA). A multi-group analysis for measurement invariance depending on being in a romantic relationship or not was assessed. The model’s reliability was also evaluated. The associations between SWLLS and CTL-I were tested by correlations, regressions and moderations. At last, differences between means and distributions concerning sociodemographic variables were determined. The results showed that a good model fit for the Portuguese version of the CTL-I was found, as well as good psychometric properties. Results also showed that satisfaction with love life contributes to explaining the capacity to love and all its dimensions, and that gender and being in a romantic relationship moderate the association between love life satisfaction and the capacity to love. Being female and being in a romantic relationship make the relationship between love life satisfaction and the capacity to love stronger and more meaningful.

## 1. Introduction

Love is inherent to the human experience, being something very intimate [1]. Hernández [2] considers that love is one of the most intense and deep emotions experienced by humans and, also, the emotion most sought after [3]. Love is a complex phenomenon that arises from a set of genetically transmitted instincts and impulses [4]. It is one of the main forces responsible for the evolution of humanity [5], being a physiological need for the survival of the individual and the species, just like food, sleep and sexuality.

Fletcher et al. [6] considered romantic love as universal; it represses mate-search mechanisms; has its own behavioral, hormonal, and neuropsychological designation; and is connected to better health and survival. Furthermore, Fletcher et al. [6] found evidence concerning relationships among mating systems, reproductive biology, and brain size. Dopamine-, vasopressin- and oxytocin-rich brain regions (in humans and other monogamous animals) orchestrate romantic love and its maintenance [7]. These authors also found polymorphisms associated with oxytocin, vasopressin and dopamine function affecting the sustainability of romantic love. Acevedo et al. [7] stated that romantic love maintenance is part of a mammalian strategy for reproduction and long-term attachment (influenced by basic reward circuitry, complex cognitive processes, and genetic factors) [7].

According to Murray et al. [8], falling in love is related to up-regulation of Type I interferon response genes, and reciprocal down-regulation of α-defensin-related transcripts. These changes are consistent with selective up-regulation of innate immune responses to viral infections and with dendritic cell facilitation of sexual reproduction [8].

Moreover, Bode and Kushnick [9] found that romantic love is a motivational state associated with a desire for long-term mating with an individual. This is associated with cognitive, emotional, behavioral, social, genetic, neural, and endocrine activity in both sexes. “Throughout much of the life course, it serves mate choice, courtship, sex, and pair-bonding functions. It is a suite of adaptations and by-products that arose sometime during the recent evolutionary history of humans” [9] (p. 21).

As a need, romantic love can be accompanied by different emotions and feelings, and can also affect reason and will [5]. According to Burunat [5], different stages of love can vary between peace, joy, jealousy, grief and sadness. Additionally, Hernández [2] states the existence of episodic manifestations of love, which vary between types and subtypes of mental events. For example, the loss of a loved one produces sadness; if it is a loss through death, mourning; the joys of the beloved tend to produce joy in us; his achievements, pride; the damage suffered by a third party, anger, etc. According to Hernández [2], these manifestations are the experience of love itself.

There are several theories of romantic love [10]) proposed by psychologists and researchers. Zick Rubin [11] differentiated between liking and loving. According to Rubin [11], romantic love includes three elements: a close bond and dependent needs, a predisposition to help, and feelings of exclusiveness and absorption. Rubin et al. [12] found that women are more prudent than men about entering into romantic relationships, more likely to compare these relationships to alternatives, more likely to end a relationship that seems perilous, and better able to cope with rejection.

Lee [13] defined six styles of love: Eros (love based on physical appearance), Storge (affection and companionship based on the idea of lasting commitment), Ludus (short-lived permissive and polygamous love, with intensity of involvement controlled and jealousy avoided), Ágape (altruistic, kind, caring, and rational love), Pragma (conscious consideration of the potential mate’s sociodemographic factors, such as education, vocation, religion and age) and Mania (based on obsession, jealousy, emotional intensity and the need for certainty that one is loved) [13]. Karandashev [14] found that people with an Eros love attitude tend to present an adaptive emotional experience and have a better chance to be happy in their romantic relationships. Ludus and Mania are maladaptive love attitudes, so that individuals with Ludus and Mania attitudes tend to be unhappy in relationships [14]. Pragma, Storge, and Agape are neutral or moderately adaptive love attitudes without intense positive and negative emotions [14].

However, the authors of the seminal work for the passionate/companionate love distinction are Walster and Walster [15] and Hatfield and Walster [16]. Romantic love is sometimes referred to as “passionate love” and differs from companionate love, which is felt less intensely and often follows a period of romantic love [16]. Elaine Hatfield [17] has described two different types of romantic love: compassionate love and passionate love. The first one is characterized by feelings of mutual respect, trust, and affection; it is a type of love that is built over time and is often seen in long-term relationships [17]. The second one involves intense feelings and sexual attraction; it is often described as a state of intense longing for union with another person [17]. Passionate love tends to be more common at the beginning of a relationship and can be associated with feelings of euphoria, excitement, and obsession [17]. Hatfield et al. [18] found that although newlywed men and women loved with equal passion, women tended to love their partners more companionately than men. The authors also found that time has a corrosive effect on love on both passionate and companionate love.

According to Sternberg [4], the author of the Triangular Theory of Love, this theory is composed of three components: intimacy, passion and commitment. Intimacy concerns closeness, connection, tenderness and bonding; passion refers to the impulses that lead to romance, physical attraction, and sexual intercourse; and commitment is characterized by the decision to love an individual and to maintain love as a form of commitment [4]. Sternberg [4], based on the three components which differ in terms of the loving experience, defines eight types of love: Non-Love (absence of the three components of love); Liking (casual and friendly relationships); Passionate Love (“love at first sight”, with passion as its main component); Empty Love (commitment and concerns relationships without emotional involvement and physical attraction); Romantic Love (intimacy and passion, therefore, individuals are emotionally connected and feel physically attracted); Companionate Love (combination of intimacy and commitment and results from a long-standing friendship); Stupid Love (association between passion and the commitment that occurs due to passion, without the presence of intimacy); and Consummate Love that includes the three components [4].

Interest in the other person and their life project constitutes a central aspect of the capacity to love, implying a mature object relationship [19]. The interest in the life of others, in their ideas, in their experience and emotional development, in their personal history and growth contribute to the enrichment and growth of one’s own life experience [19]. Basic trust, gratitude for the existence of the loved one and humility in accepting the other’s needs belongs to the characteristics of the ability to love [19]. This provokes a sense of responsibility for the other, their happiness, the realization of their life project as a personal goal [19]. In a mature love relationship, the idealization of the other’s body, their physical beauty, the changing aspects of the body, due to aging or illness, should not affect love [19].

In fact, individuals present the need to feel loved and safe [20]; however, Josselyn [20] considers the existence of a very limited capacity to love others. Indeed, the ability to love implies care and trust in others [21]; the existence of mutuality within the relationship implies the ability to search for love and, equally, the ability to love [22]. Based on Diener and colleagues’ components of the satisfaction with life [23], Neto [24] developed an instrument to measure love life satisfaction. According to Neto [24], love satisfaction may be predicted by other aspects of romantic relationships, namely, marital quality and stability. However, love satisfaction may also explain the capacity to love, especially if the relation is moderated by other variables such as gender or being or not in a romantic relationship. Thus, this study aims to evaluate if the relation between love life satisfaction, assessed by the satisfaction with love life scale (SWLLS), and capability to love, assessed by the capacity to love inventory (CTL-I), is moderated by gender and romantic relationship in a Portuguese sample. We expect to find a good model fit for the Portuguese version of the CTL-I. We also expect to find invariance of the CTL-I depending on being in a romantic relationship or not. We suppose that we will find good values of model reliability. We also expect to find associations between the CTL-I and the SWLLS. At last, we expect to find differences between means and distributions concerning sociodemographic variables.

## 2. Methods

### 2.1. Procedures

All procedures took into consideration the Helsinki Declaration [25] for human research, as well as its most recent updates. This study was approved by the Scientific Council of the Universidade Católica Portuguesa. Initially, authorization was requested from the authors of the original version of the CTL-I instrument. The translation and back-translation of the CTL-I from English to Portuguese, according to the international guidelines [26], were carried out by two native English and one Portuguese psychologist. Then, a protocol that includes an informed consent, a sociodemographic questionnaire, the CTL-I and SWLLS instruments was conceived. The informed consent contained an explanation regarding the theme and aims of the study and the voluntary nature of the participants’ attendance. Participants were also informed that they could withdraw at any time, without any type of cost or risk. This informed consent ensured complete confidentiality and privacy of their data. The inclusion criteria were having Portuguese nationality, being a native Portuguese, being over 18 years old and signing the informed consent form. The exclusion criteria included not meeting the inclusion criteria and not completing the entire survey. Incomplete questionnaires were removed. The research protocol was made available online on Google Forms and disseminated through social media. Data were collected between 14 February and 23 April, 2023 based on informal contacts and using the snowball technique [27].

### 2.2. Participants

This study used a total sample (*N* = 1017); this sample was divided into two parts for the purposes of validating the CTL-I: a sample intended for exploratory factor analysis (*N* = 508) and another for confirmatory factor analysis (*N* = 509). Thus, the total sample included 1017 participants, of which 830 were women (81.6%) aged from 18 to 72 years old (*M* = 32.44; *SD* = 12.14). Practically half of the sample went to university (531 or 52.2%) and the other half did not (486 or 47.8%). The majority of the sample was single (607 or 59.7%), 324 or 31.9% were married and 86 or 8.5% were separated, divorced or widowed. More than half of the sample (754 or 74.1%) was in a romantic relationship.

There were no statistically significant differences between the two samples concerning gender (χ^2^ = 0.304; *p* = 0.581), age (*t* = 0.929; *p* = 0.353), education (χ^2^ = 0.283; *p* = 0.595), marital status (χ^2^ = 4.269; *p* = 0.118), and being in a romantic relationship or not (χ^2^ = 0.233; *p* = 0.629).

### 2.3. Measures

#### 2.3.1. Sociodemographic Questionnaire

The sociodemographic questionnaire included the following questions: gender (0—Male and 1—Female), age, educational qualifications (0—did not go to university; 1—went to university), marital status (0—Single; 1—Married; 2—Widowed; legally separated and divorced) and romantic relationship (0—No and 1—Yes). In the last question, if the answer was negative, the participant was instructed to take into consideration a previous relationship in order to answer the instruments correctly.

#### 2.3.2. Capacity to Love-Inventory (CTL-I)

The Capacity to Love-Inventory (CTL-I) was developed by Kapusta and colleagues [28] to assess the ability to love as a personality trait, taking into account the individual’s perception of their partner [28]. Initially, this instrument consisted of 70 items, which were later reduced to 41 [28]. The dimensions of the scale are Interest in the Other’s Life Project (INT), which includes items 1 to 7, with item 7 being inverted; Basic Trust (BTR), which includes items 8 to 16, with items 13 and 15 being reversed; Gratitude (GRT) covers items 17 to 23; the Common Ideal Ego (CEI), which includes items 24 to 31; Permanence of Sexual Passion (PSP) comprises items 32 and 33, both of which are inverted, and Loss and Grief (LOM), with items 34 to 41, all inverted [28]. The CTL-I items were evaluated using a four-point Likert scale, ranging between 1 and 4, with the response modalities being as follows: 1 (definitely no), 2 (probably no), 3 (maybe yes) and 4 (definitely yes) [28]. A high score suggests a greater capacity to love. Regarding psychometric properties, the CTL-I presents good internal consistency, with stable and consistent results in two culturally different samples, and broad test–retest reliability [28]. The six dimensions of the CTL-I show moderate correlations with each other. The Cronbach’s alpha for the CTL-I total was α = 0.90 [28]. There are some versions of this instrument: the original one, which is Austrian [28] and Polish [28], Italian [29], Chinese [30], and Slovenian [31].

#### 2.3.3. Satisfaction with Love Life (SWLLS)

The Satisfaction with Love Life scale (SWLLS) was developed by Neto [24] and aims to assess the satisfaction that individuals feel with their love life [24]. This love life is related to actual “love” rather than simply romantic relationships. According to Neto [24], love life satisfaction refers to a cognitive, judgmental process, in which individuals assess their love lives. The SWLLS was developed based on the original SWLS [23,32]. The SWLLS is unifactorial and is composed of 5 items, which are evaluated on a 7-point Likert scale, ranging between 1 and 7 [24]: 1 (completely disagree), 2 (disagree), 3 (more or less disagree), 4 (neither agree nor disagree), 5 (more or less agree), 6 (agree) and 7 (totally agree) [24]. A high score indicates a greater satisfaction with the love life. The Cronbach’s alpha obtained was α = 0.91 [24].

#### 2.3.4. Data Analysis

To achieve the main aim of this study, CTL-I was adapted and validated for this population. Measurement invariance across gender and depending on being in a romantic relationship or not was assessed. The model reliability was also evaluated. Then, the associations between CTL-I and SWLLS were tested by correlations, regressions and moderations. At last, the differences between means and distributions concerning sociodemographic variables were determined.

Preliminary analyses were carried out to assess (1) the normality of the items through skewness (SI < 2) and kurtosis (KI < 10), (2) multicollinearity, which was checked by the tolerance (>0.100) and variance inflation factor (VIF) (<10) (*r*  > 0.80) [33], and (3) the items’ description, as well as the scale mean if the item is deleted, the scale variance if the item is deleted, the corrected total item correlation, and Cronbach’s alpha if the item is deleted.

The exploratory factor analysis (EFA) (maximum likelihood) with the principal component analysis (PCA) were conducted for the 35 items by running an orthogonal (i.e., Varimax) rotated analysis in order to achieve a factor structure for these variables. Sample adequacy was assessed using Kaiser-Meyer-Olkin (KMO) value [34] and Bartlett’s Sphericity [35]. Factors were assessed using eigenvalues greater than 1 [36]. Items were removed based on communalities (<0.30), factor loadings (<0.40), and if Cronbach’s alpha increased if item deleted.

The assumptions of a CFA include multivariate normality, a sufficient sample size (n > 200), the correct a priori model specification, and data must come from a random sample. To assess the adequacy of the instruments and the goodness of fit, Robust Maximum Likelihood (MLR) confirmatory factor analyses (CFAs) were carried out. To evaluate the CFA models, the root mean square error of approximation (RMSEA), the comparative and incremental fit indices (CFI and IFI, respectively), Tucker–Lewis index (TLI), the goodness of fit (GFI), the standardized root mean square residual (SRMR) and the Akaike information criterion (AIC) were taken into account. When the CFI, the IFI, the TLI and GFI were ≥0.95, the RMSEA ≤ 0.05, and the SRMR ≤ 0.05 [37], a very good model fit is identified. When values ≥0.90 for the CFI and the IFI, ≤0.08 for the RMSEA, and ≤0.10 for the SRMR are found, an acceptable model is identified [38]. It also reported the Satorra–Bentler chi-square (χ^2^), general model significance (*p*), and relative chi-square (χ^2^/df).

Multi-group CFAs were carried out to evaluate if the factor structure of the scales were valid for their use depending on them being in in a romantic relationship or not: four levels of measurement invariance were tested: configural (items load on the same factor across groups); metric (item factorial loadings are equal across groups); scalar (item intercepts are equal across groups) and error variance invariance (items measurement error are equal across groups). Through the difference between pairs of nested models (Δ) in the RMSEA, CFI and SRMR, the progressive constrained models were evaluated. A change ≥0.01 in the CFI, ≥0.015 in the RMSEA, and ≥0.03 in the SRMR suggests a statistically significant decrease in the model fit when testing for measurement invariance [39].

Pearson correlations for continuous variables and Spearman correlations when at least one of the variables was ordinal or nominal were established, meaning that the correlation values were classified as between 0 and 0.3 being weak, between 0.3 and 0.5 being moderate, between 0.5 and 0.7 being strong, and between 0.7 and 1 being very strong, either positive or negative [40]. To assess the model reliability, convergent and discriminant validity, Cronbach’s alpha coefficients, MacDonald’s Omega, composite reliability (CR, 0.70 or higher suggests good model reliability), average variance extracted (AVE, 0.50 or higher suggests adequate convergence) and square root of AVE (higher than the highest correlation with any other latent variable) were assessed.

Multiple linear regressions were carried out to assess the variables that predicted the capacity to love and its subscales. Also, simple moderations were performed to assess the moderating role of gender and being in a relationship, in the association between love life satisfaction and capacity to love.

T-test independent means was applied to compare the means of two groups. F-test was used to compare the means of more than two groups. Cohen’s d effect-size and eta squared effect-size were used accordingly to the level of measurement of the variables [41]. The statistical significance level was set at 0.05. Statistical analysis was performed using SPSS version 28, PROCESS and AMOS version 28.

## 3. Results

### 3.1. Preliminary Analyses (N = 1017)

Capacity to Love Inventory items present skewness and kurtosis scores within the reference values, thus ensuring their normal distribution, except for items 3 and 4. Also, tolerance and VIF are within the reference values ensuring the absence of multicollinearity. Furthermore, the Cronbach’s alpha value of the total scale increases slightly if some items are removed, namely, items 13, 24, 38, 39, 40, and 41 (Table 1).

### 3.2. Adaptation and Validation of the Capacity to Love Inventory (CTL-I) for the Portuguese Population

#### 3.2.1. EFA (*N* = 508)

An exploratory factorial analysis was carried out, without determination of the factors numbers, and it found a structure in which the 41 items were distributed by seven factors, explaining 58.26% of the total variance. Although most indicators are within reference values, items 5, 6, 7, 9, 12, 15, 25, 28, and 30 were not discriminatory, as they saturated very closely in more than two factors; thus, it was decided to exclude them (Table 2). Moreover, in the seventh factor, only one item (item 13) saturated properly. We repeated the exploratory factor analysis without the nine items identified above and, once again, the resulting structure included 32 items distributed across six factors, explaining 60.71% of the total variance. Most indicators are within reference values; however, item 13 presented a very low communality, so it was decided to exclude it. After repeating the analysis without item 13, a structure of 31 items grouped into six factors was found, explaining 62.28% of the total variance (Table 3).

#### 3.2.2. CFA (*N* = 509)

**Figure 1:**
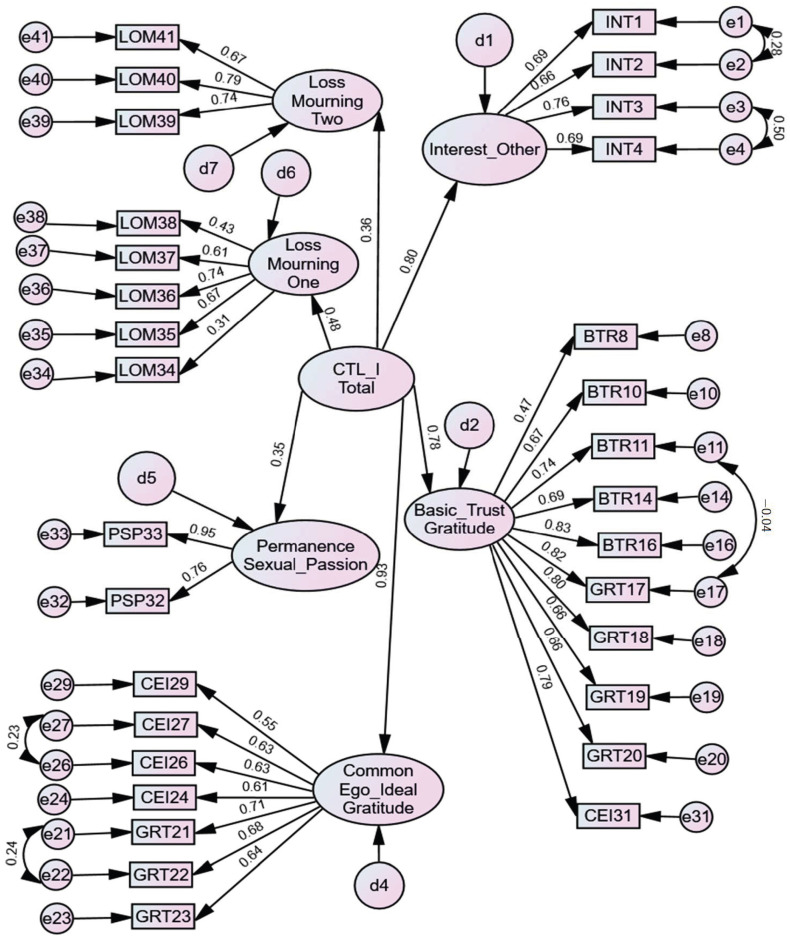
The final CFA model of CTL-I.

The assumptions of the CFA were met. After the EFA, a confirmatory factor analysis (CFA) was carried out with the aim of confirming the model found in the EFA. The initial model found (seven factors because the 7th factor contained only one item) did not present a good fit [χ^2^(433) = 3.784; CFI = 0.837; TLI = 0.835; IFI = 0.837; GFI = 0.830; RMSEA = 0.074 (CI 0.070–0.078); SRMR = 0.087; AIC = 1764.580]. Modification indices were used and suggested correlations between some items (Figure 1), theoretically supported, were established, and then a good model was found [χ^2^(402) = 2.228; CFI = 0.930; TLI = 0.919; IFI = 0.931; GFI = 0.900; RMSEA = 0.050 (CI 0.046–0.055); SRMR = 0.067; AIC = 1105.470]. Finally, the scale model comprises 31 items distributed across six factors. Factor one includes items 8, 10, 11, 14, 16, 17, 18, 19, 20 and 31. Factor two contains items 21, 22, 23, 24, 26, 27, and 29. Factor three has items 1, 2, 3, and 4. Factor four includes items 39, 40, and 41. Factor five contains items 34, 35, 36, 37, and 38. Finally, factor six includes items 32 and 33.

### 3.3. Measurement of Invariance

Results of the multi-group analysis for measuring the invariance of the CTL-I depending on being in in a romantic relationship or not are presented in Table 4. The configural invariance according to being in a romantic relationship or not was confirmed during the first step of the multi-group CFAs. The same happens with metric, scalar and error variance invariance. The results provide evidence of invariance in relation to CTL-I when it comes to being in a romantic relationship or not (Table 4).

### 3.4. Model Reliability

The reliability indices for the CTL-I factors are displayed in Table 5. All the correlations between the CTL-I dimensions were positive and statistically significant, except interest in others that does not correlate with loss and mourning, and common ego ideal that does not correlate with mourning. No significant differences between Cronbach’s alpha (α) and McDonald’s omega (ω) were observed, except for factor 4, for which the McDonald’s omega could not be calculated because this factor only contains two items. The values of Cronbach’s alpha and McDonald’s omega are within the acceptable limit. Thus, the CTL-I is a reliable measure. Additionally, composite reliability, average variance extracted (AVE), square root of AVE, mean and standard deviation were calculated (Table 5), and all the values were within the reference range.

After analyzing the content of the items for each factor, they were designated as follows: Factor 1 is interest in other, factor 2 is basic trust and gratitude, factor 3 is common ego ideal, factor 4 is permanence of sexual passion, factor 5 and factor 6 correspond to the last factor of the original version and they are designated as factor 5 being loss, and factor 6 being mourning.

### 3.5. Assessing the Satisfaction with Love Life Scale (SWLLS)

#### 3.5.1. CFA

**Figure 2:**
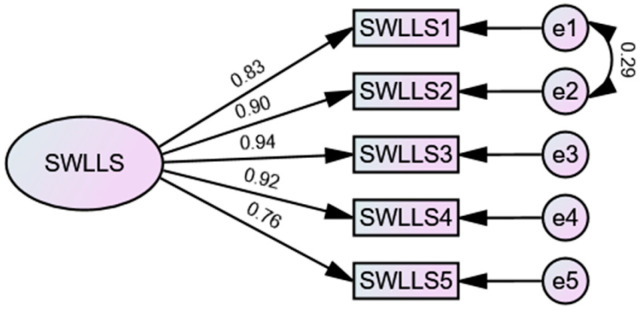
The CFA model of SWLLS.

SWLLS items present skewness and kurtosis scores within the reference values, thus ensuring their normal distribution. Also, tolerance and VIF are within the reference values ensuring the absence of multicollinearity. The assumptions of the CFA were met The fit of the SWLLS to the sample is very good [χ^2^(4) = 4.679; CFI = 0.997; TLI = 0.992; IFI = 0.997; GFI = 0.993; RMSEA = 0.060 (CI 0.034–0.089); SRMR = 0.008; AIC = 40.718] (Figure 2).

#### 3.5.2. Model Reliability

The reliability indices for the SWLLS were: Cronbach’s alpha 0.938; McDonald’s omega 0.940. Besides, composite reliability (0.956), average variance extracted (AVE) (0.814), square root of AVE (0.902), mean (5.03) and standard deviation (1.65) were calculated and all the values were above the reference range. Thus, the SWLLS is a reliable measure.

### 3.6. Associations between CTL-I and SWLLS

#### 3.6.1. Correlations

The SWLLS correlates positively and significantly with the CTL-I total and with all the CTI-L subscales: CTL-I total (*r* = 0.645; *p* < 0.001); interest in others (*r* = 0.280; *p* < 0.001); basic trust and gratitude (*r* = 0.659; *p* < 0.001); common ego ideal (*r* = 0.413; *p* < 0.001); permanence sexual passion (*r* = 0.238; *p* < 0.001); loss (*r* = 0.255; *p* < 0.001); and mourning (*r* = 0.286; *p* < 0.001).

#### 3.6.2. Multiple Linear Regressions

Aiming to determine the contribution of sociodemographic variables and the SWLLS variable to explain the CTL-I and its dimensions, a set of multiple linear regressions was carried out. SWLLS contributes strongly to explain the CTL-I total and all its dimensions. Age also contributes to explain the CTL-I total and all its dimensions, except loss. Being in a romantic relationship also contributes to explain the CTL-I total and three dimensions. The same happens with gender. Education only contributes to explain two subscales (Table 6).

#### 3.6.3. Moderations

To assess the moderating role of being or not in a romantic relationship and of gender in the relation between SWLLS and CTL-I, several moderations were carried out. Our results showed that a romantic relationship moderates the relation between SWLLS, for one hand, and the CTL-I total, interest in others, basic trust and gratitude, common ego ideal, and permanent sexual passion, on another hand, when the moderator variable is “yes” (Table 7). Also, gender moderates the relationship between SWLLS, on the one hand, and the CTL-I total and basic trust and gratitude, when the moderator variable (gender) is female (Table 7).

#### 3.6.4. Differences

Concerning gender, there are statistically significant differences in relation to the CTL-I subscale, interest in others [*t*(220, 196) = −3.254; *p* < 0.001; *d* = −0.349 (−0.508–−0.190)], with women (*M* = 3.86, *SD* = 0.30) scoring higher than men (*M* = 3.74, *SD* = 0.48). Regarding education, there are statistically significant differences in relation to the CTL-I subscale, basic trust and gratitude [*t*(1014, 215) = 2.180; *p* = 0.029; *d* = 0.136 (0.013–0.259)], with participants who did not go to university (*M* = 3.55, *SD* = 0.52) scoring higher than participants who went to university (*M* = 3.47, *SD* = 0.59). The same happens with CTL-I subscale permanence sexual passion [*t*(1014, 1051) = 4.895; *p* < 0.001; *d* = 0.306 (0.183–0.430)], with participants who did not go to university (*M* = 2.97, *SD* = 0.88) scoring higher than participants who did not go to university (*M* = 2.69, *SD* = 0.93). Furthermore, the same happens with SWLLS [*t*(1014, 937) = 2.753; *p* = 0.006; *d* = 0.172 (0.049–0.295)], with participants who did not go to university (*M* = 5.17, *SD* = 1.58) scoring higher than participants who went to university (*M* = 4.89, *SD* = 1.71). There are statistically significant differences between the CTL-I (except for the loss subscale) and SWLLS depending on the marital status (Table 8). The post hoc Tuckey test shows that the differences mainly occur between the single and the separated, divorced, and widower groups and between married or in a relationship and separated, divorced, and widower groups, being that this last group generally presents the lower values and the married or in a relationship the higher values. The exceptions are in permanence sexual passion (the higher values appear in singles) and in mourning, in which the higher values appear in the separated, divorced, and widower groups.

Age correlates with almost all the dimensions studied: CTL-I total (*r* = −0.144; *p* < 0.001); basic trust and gratitude (*r* = −0.192; *p* < 0.001); common ego ideal (*r* = −0.157; *p* < 0.001); permanence sexual passion (*r* = −0.300; *p* < 0.001); mourning (*r* = 0.194; *p* < 0.001); and SWLLS (*r* = −0.072; *p* = 0.021).

## 4. Discussion

This study aimed to evaluate if the relation between satisfaction with the love life and capacity to love is moderated by gender and by being in a romantic relationship or not in a Portuguese sample. To this end, it was carried out the validation of CTL-I [28] for this population and a good model fit for the Portuguese version of the CTL-I was found, as well as good psychometric properties. The Portuguese version of the CTL-I includes 31 items distributed by six factors. The first one, interest in others, maintained four of the original seven items [28]. The second factor, basic trust and gratitude, includes five items of the original, four items of the original third factor, gratitude and one item of the original fourth factor, common ego ideal. The third factor of this study, the common ego ideal, includes four items of the original fourth factor (common ego ideal) and three items of the original third factor (gratitude). Permanence of sexual passion keeps items 32 and 33. At last, this study’s final factors, loss (fifth), and mourning (sixth), respectively, divide the items of the original sixth factor (loss and mourning). The reorganization of items in this study was unparalleled in other validations of the CTL-I [29,30,31]. In fact, the Italian and the Slovenian CTL-I fully replicated the six-factor structure of the original CTL-I [28]. All versions presented good psychometric qualities. However, the content of the items theoretically justifies the reorganization of the items in the present study, despite having maintained almost all of the names of the original factors. This study demonstrated that the six-factor structure of the original inventory was not supported by the data. Furthermore, this study theoretically derived a six-factor model, with rearrangement of the new items, through EFA and CFA. This model showed that the new proposed six-factor structure is a better fit to the data, having greater statistical validity [42,43].

Love life satisfaction correlates positively and significantly with the capacity to love (total) and all its dimensions; the highest values of these correlations occurred between satisfaction with love life, on the one hand, and capacity to love (total) and basic trust and gratitude on the other hand. These results corroborate those of Rubin [11], who stated that romantic love includes a close bond and dependent needs and a predisposition to help.

Love life satisfaction contributes to explain the capacity to love (total) and all its dimensions. Moreover, being in a relationship contributes to the capacity to love and its dimensions. In fact, Busch and Kapusta [44] found that single women, when compared to women who are in a relationship, have a lower capacity to love and suffer more from depressive symptoms. This study’s results are in line with those of [45] Hudson et al. (2020), who stated that being in a romantic relationship predicts greater well-being, although the effects were moderated by relationship quality. Also, Gómez-López et al. [46] found that romantic relationships are a predictor of psychological well-being, linked to positive interpersonal relationships and life development.

Being in a romantic relationship moderates the relation between satisfaction with love life, on the one hand, and capacity to love (total), interest in others, basic trust and gratitude, common ego ideal, and permanence sexual passion, on the other hand. It means that being in a romantic relationship enhances the association between love life satisfaction and capacity to love [47]. Hudson et al. [45] found that romantic relationship quality is associated with well-being.

Additionally, being female moderates the relationship between satisfaction with love life and capacity to love (total), and basic trust and gratitude. This result is interesting because neither Neto [24], nor Neto and Pinto [48] found gender differences in love life satisfaction nor this has been found in this study.

This study has some limitations that must be addressed, namely the fact that the sample is not representative of the Portuguese population; this can be taken into account in future studies. The fact that it was not possible to confirm the model proposed by the original authors of CTL-I requires further studies with more representative samples of the Portuguese population, on the one hand; and by more specific samples within the population (e.g., youth and young adults), on the other hand. Furthermore, the size of the inventory (CTL-I) limited the confirmation of the authors’ model, given its complexity. Therefore, more studies are needed to confirm the Portuguese version.

Although the values of the Composite Reliability, AVE and SQRT-AVE suggest good divergent and convergent validity, the absence of in-depth analyses of convergent and divergent validity (with instruments measuring similar and different constructs) is a limitation and should be addressed in future studies.

## 5. Conclusions

The novelty of this study lies in the result that love life satisfaction predicts the capacity to love. Some authors [49,50,51] have found an inverse relationship (the capacity to love predicts life satisfaction); however, the reciprocity of this relationship had not been tested. In fact, if someone feels satisfaction in relation to their romantic life, this could translate into an increase or improvement in their capacity to love. Other novelty of this study is related with the moderating role of gender and of being in a romantic relationship in the association between love life satisfaction and capacity to love. Being a woman and being in a romantic relationship intensifies the predictive power of love life satisfaction in relation to the capacity to love.

## Figures and Tables

**Table 1:** Descriptive statistics of the Capacity to Love Inventory (CTL-I) items.

Item	*M*	*SD*	σ^2^	Sk (*SD* 0.08)	β^2^ (*SD* 0.15)	±	VIF	Scale Mean If Item Deleted	Scale Variance If Item Deleted	Corrected Total Item Correlation	α If Item Is Deleted
Total	3.72	0.36	0.13	−1.07	1.87						0.915 (total)
CTL_I1	3.80	0.47	0.22	−2.61	8.31	0.54	1.87	134.46	215.21	0.37	0.914
CTL_I2	3.75	0.51	0.26	−2.16	5.35	0.47	2.12	134.51	212.85	0.50	0.913
CTL_I3	3.91	0.35	0.12	−5.00	29.45	0.40	2.50	134.35	215.84	0.44	0.914
CTL_I4	3.90	0.40	0.16	−5.16	30.14	0.51	1.98	134.35	215.82	0.39	0.914
CTL_I5	3.40	0.77	0.59	−1.24	1.19	0.78	1.29	134.86	213.71	0.28	0.915
CTL_I6	3.63	0.59	0.35	−1.77	4.07	0.64	1.56	134.63	212.73	0.43	0.913
CTL_I7	2.95	0.83	0.69	−0.46	−0.36	0.68	1.46	135.30	209.74	0.42	0.914
CTL_I8	3.25	0.80	0.65	−0.98	0.58	0.63	1.58	135.01	208.89	0.47	0.913
CTL_I9	3.24	0.79	0.62	−0.86	0.29	0.60	1.67	135.02	207.74	0.54	0.912
CTL_I10	3.47	0.75	0.56	−1.37	1.43	0.36	2.81	134.79	205.64	0.67	0.911
CTL_I11	3.52	0.75	0.56	−1.60	2.10	0.33	3.08	134.74	205.40	0.68	0.910
CTL_I12	3.67	0.57	0.33	−1.78	3.39	0.53	1.91	134.59	210.54	0.58	0.912
CTL_I13	3.12	1.02	1.03	−0.72	−0.82	0.79	1.27	135.14	210.61	0.30	0.916
CTL_I14	3.50	0.72	0.52	−1.43	1.69	0.43	2.33	134.76	206.47	0.66	0.911
CTL_I15	2.92	1.01	1.02	−0.40	−1.07	0.65	1.54	135.34	205.26	0.49	0.913
CTL_I16	3.67	0.61	0.37	−2.08	4.67	0.33	3.07	134.59	207.71	0.71	0.911
CTL_I17	3.71	0.61	0.37	−2.37	5.93	0.30	3.35	134.55	208.36	0.68	0.911
CTL_I18	3.65	0.67	0.45	−2.13	4.45	0.32	3.17	134.61	207.05	0.68	0.911
CTL_I19	3.46	0.76	0.57	−1.38	1.43	0.46	2.17	134.80	206.98	0.60	0.911
CTL_I20	3.39	0.80	0.64	−1.22	0.88	0.49	2.06	134.87	207.28	0.55	0.912
CTL_I21	3.80	0.47	0.22	−2.69	8.92	0.40	2.52	134.46	212.27	0.59	0.913
CTL_I22	3.81	0.45	0.21	−2.73	8.89	0.43	2.34	134.45	213.15	0.54	0.913
CTL_I23	3.62	0.59	0.35	−1.52	2.57	0.56	1.80	134.64	211.15	0.53	0.913
CTL_I24	3.72	0.54	0.29	−2.04	4.45	0.56	1.78	134.54	212.78	0.48	0.913
CTL_I25	3.43	0.75	0.56	−1.25	1.15	0.40	2.49	134.83	205.79	0.67	0.911
CTL_I26	3.61	0.58	0.34	−1.49	2.57	0.51	1.95	134.65	210.67	0.57	0.912
CTL_I27	3.66	0.58	0.34	−1.78	3.44	0.54	1.87	134.60	211.45	0.52	0.913
CTL_I28	3.52	0.78	0.61	−1.72	2.40	0.64	1.58	134.74	209.54	0.46	0.913
CTL_I29	3.63	0.58	0.34	−1.46	2.06	0.58	1.74	134.63	211.31	0.53	0.913
CTL_I30	3.30	0.89	0.80	−1.10	0.25	0.59	1.68	134.96	206.11	0.53	0.912
CTL_I31	3.44	0.77	0.60	−1.40	1.55	0.29	3.51	134.81	203.61	0.74	0.910
CTL_I32	2.78	0.99	0.97	−0.21	−1.07	0.45	2.23	135.48	209.86	0.34	0.915
CTL_I33	2.86	1.00	1.01	−0.34	−1.06	0.44	2.29	135.40	209.09	0.36	0.915
CTL_I34	2.08	0.93	0.87	0.55	−0.54	0.81	1.23	136.18	215.27	0.16	0.917
CTL_I35	3.25	0.78	0.60	−0.81	0.17	0.72	1.40	135.01	213.66	0.28	0.915
CTL_I36	3.28	0.89	0.79	−0.97	−0.10	0.66	1.51	134.98	210.41	0.36	0.914
CTL_I37	3.21	0.91	0.83	−0.84	−0.37	0.64	1.58	135.05	211.45	0.31	0.915
CTL_I38	2.80	0.91	0.83	−0.25	−0.82	0.77	1.30	135.46	216.71	0.11	0.918
CTL_I39	2.87	0.96	0.92	−0.29	−1.02	0.58	1.73	135.39	212.76	0.25	0.916
CTL_I40	2.94	1.03	1.07	−0.42	−1.14	0.51	1.94	135.32	210.33	0.31	0.916
CTL_I41	2.75	1.02	1.04	−0.19	−1.14	0.59	1.69	135.51	214.28	0.18	0.918

*M* = mean; *SD* = standard deviation; σ^2^ = variance; Sk = skewness; β^2^ = kurtosis; ± = tolerance; VIF = variance inflation factor; α = Cronbach’s alpha.

**Table 2:** Exploratory factor analysis of the Capacity to Love-Inventory (CTL-I) (initial 41 items).

Item	*h^2^*	*LD1*	*LD2*	*LD3*	*LD4*	*LD5*	*LD6*	*LD7*
CTL_I1	0.61	0.22	0.21	**0.72**	−0.02	0.01	0.01	−0.05
CTL_I2	0.63	0.42	0.24	**0.62**	−0.03	−0.05	0.03	0.12
CTL_I3	0.70	0.21	0.29	**0.75**	−0.03	0.01	−0.05	0.03
CTL_I4	0.56	0.20	0.16	**0.70**	−0.06	0.03	−0.01	0.06
CTL_I5	0.40	0.09	0.20	0.37	0.05	−0.09	−0.01	0.45
CTL_I6	0.54	0.18	0.34	0.41	0.04	0.17	−0.07	0.44
CTL_I7	0.46	0.42	0.17	−0.21	−0.10	0.34	0.21	0.18
CTL_I8	0.44	**0.60**	0.07	0.07	−0.07	0.04	0.04	0.24
CTL_I9	0.48	0.41	0.07	0.29	−0.03	0.02	0.21	0.43
CTL_I10	0.66	**0.73**	0.18	0.19	0.06	0.06	0.14	0.20
CTL_I11	0.73	**0.78**	0.20	0.21	0.04	0.07	0.09	0.15
CTL_I12	0.51	0.34	0.31	0.40	0.03	0.05	0.08	0.35
CTL_I13	0.49	0.13	0.14	−0.09	0.04	0.10	0.09	**0.66**
CTL_I14	0.59	**0.64**	0.24	0.19	0.00	0.09	0.16	0.21
CTL_I15	0.42	0.38	0.06	−0.08	0.33	0.13	0.18	0.33
CTL_I16	0.70	**0.73**	0.22	0.31	0.03	0.08	0.11	0.04
CTL_I17	0.72	**0.70**	0.35	0.31	0.08	0.02	−0.02	−0.08
CTL_I18	0.75	**0.73**	0.34	0.29	0.03	0.07	0.01	−0.09
CTL_I19	0.61	**0.72**	0.24	0.16	0.07	0.00	−0.05	0.02
CTL_I20	0.53	**0.63**	0.34	0.04	0.05	−0.10	−0.07	0.06
CTL_I21	0.63	0.39	**0.63**	0.28	−0.04	−0.04	0.00	0.02
CTL_I22	0.60	0.36	**0.60**	0.33	−0.02	0.00	0.01	0.06
CTL_I23	0.57	0.35	**0.62**	0.13	−0.03	0.02	−0.10	0.18
CTL_I24	0.60	0.13	**0.72**	0.07	0.05	−0.12	0.10	0.19
CTL_I25	0.64	0.54	0.55	0.08	−0.01	0.09	0.15	−0.03
CTL_I26	0.55	0.20	**0.66**	0.17	−0.02	0.19	0.08	0.07
CTL_I27	0.55	0.29	**0.64**	0.22	−0.05	0.02	0.06	0.03
CTL_I28	0.47	0.41	0.47	0.23	0.10	−0.11	−0.05	0.09
CTL_I29	0.50	0.22	**0.61**	0.18	−0.01	0.08	0.04	0.19
CTL_I30	0.45	0.40	0.49	0.11	0.06	−0.04	0.17	0.00
CTL_I31	0.77	**0.75**	0.40	0.14	0.04	−0.02	0.12	0.10
CTL_I32	0.75	0.10	0.06	0.01	0.03	0.14	**0.84**	0.11
CTL_I33	0.82	0.14	0.11	−0.03	0.00	0.06	**0.88**	0.05
CTL_I34	0.34	0.15	−0.18	−0.06	0.18	**0.44**	0.23	0.00
CTL_I35	0.55	−0.04	0.09	0.04	0.02	**0.69**	0.24	−0.03
CTL_I36	0.50	0.02	0.17	0.08	0.18	**0.65**	0.02	0.09
CTL_I37	0.51	0.05	−0.06	0.05	0.43	**0.55**	0.08	0.01
CTL_I38	0.54	−0.01	−0.09	−0.04	0.15	**0.66**	−0.26	0.09
CTL_I39	0.67	0.01	0.09	−0.08	**0.80**	0.13	−0.01	0.02
CTL_I40	0.72	0.12	−0.02	−0.02	**0.82**	0.20	0.01	0.02
CTL_I41	0.65	−0.04	−0.06	0.02	**0.79**	0.14	−0.01	0.04
Eigenvalues		12.84	3.43	2.10	1.69	1.49	1.21	1.12
Total variance explained		31.31%	8.37%	5.12%	4.13%	3.64%	2.96%	2.73%
Correlation matrix		[0.30–0.90]	0.100–0.675					
Determinant score		[>0.00001]	4.557 × 10^−10^					
Bartlett’s Test of Sphericity (df); *p* < 0.05					10,591.14 (820); *p* < 0.001			
Kaiser-Meyer-Olkin Measure (KMO) (above 0.50)					0.955			
Diagonal element anti-correlation matrix (above 0.50)					0.70–0.98			
Cronbach’s alfa (α) (>0.70) 0.928			0.865	0.818	0.784	0.656	0.822	-

*h^2^* = extracted communality coefficients; *LD* = structure coefficients; **bold** = items’ saturation; grey = excluded items.

**Table 3:** Exploratory factor analysis of the Capacity to Love-Inventory (CTL-I) (final 31 items).

Item	*h^2^*	*LD1*	*LD2*	*LD3*	*LD4*	*LD5*	*LD6*
CTL_I1	0.64	0.23	0.19	**0.74**	−0.02	0.01	0.00
CTL_I2	0.64	0.43	0.25	**0.62**	−0.03	−0.04	0.03
CTL_I3	0.73	0.23	0.27	**0.78**	−0.01	0.00	−0.06
CTL_I4	0.61	0.20	0.15	**0.74**	−0.05	0.01	−0.01
CTL_I8	0.44	**0.64**	0.10	0.00	−0.08	0.06	0.08
CTL_I10	0.64	**0.74**	0.19	0.17	0.07	0.06	0.15
CTL_I11	0.73	**0.80**	0.18	0.20	0.05	0.06	0.12
CTL_I14	0.58	**0.67**	0.27	0.15	0.00	0.10	0.17
CTL_I16	0.71	**0.76**	0.17	0.29	0.03	0.07	0.13
CTL_I17	0.69	**0.72**	0.27	0.32	0.08	0.01	−0.03
CTL_I18	0.72	**0.75**	0.26	0.29	0.03	0.04	0.00
CTL_I19	0.62	**0.75**	0.18	0.13	0.06	0.00	−0.04
CTL_I20	0.54	**0.66**	0.30	0.02	0.03	−0.08	−0.07
CTL_I21	0.66	0.42	**0.64**	0.25	−0.02	−0.06	−0.01
CTL_I22	0.63	0.41	**0.62**	0.28	−0.01	−0.01	0.01
CTL_I23	0.57	0.40	**0.63**	0.10	−0.04	0.03	−0.09
CTL_I24	0.61	0.16	**0.74**	0.08	0.06	−0.13	0.10
CTL_I26	0.53	0.24	**0.65**	0.15	0.00	0.15	0.10
CTL_I27	0.55	0.32	**0.63**	0.22	−0.05	0.01	0.06
CTL_I29	0.52	0.26	**0.64**	0.17	−0.01	0.09	0.06
CTL_I31	0.76	**0.78**	0.36	0.13	0.04	−0.01	0.12
CTL_I32	0.78	0.12	0.08	−0.02	0.03	0.14	**0.86**
CTL_I33	0.83	0.15	0.09	−0.03	0.00	0.05	**0.89**
CTL_I34	0.35	0.15	−0.18	−0.09	0.16	**0.46**	0.23
CTL_I35	0.57	−0.04	0.09	0.05	−0.02	**0.71**	0.24
CTL_I36	0.52	0.04	0.20	0.06	0.16	**0.67**	0.02
CTL_I37	0.52	0.06	−0.07	0.04	0.41	**0.58**	0.09
CTL_I38	0.53	0.00	−0.06	−0.07	0.14	**0.67**	−0.24
CTL_I39	0.69	0.01	0.09	−0.08	**0.81**	0.13	0.00
CTL_I40	0.73	0.12	−0.03	−0.04	**0.82**	0.22	0.02
CTL_I41	0.67	−0.03	−0.06	0.02	**0.80**	0.15	0.00
Eigenvalues		10.12	3.19	1.89	1.60	1.38	1.13
Total variance explained		32.64%	10.28%	6.09%	5.17%	4.44%	3.65%
Correlation matrix [0.30–0.90]		0.100–0.675					
Determinant score [>0.00001]		6.676 × 10^−8^					
Bartlett’s Test of Sphericity (df); *p* < 0.05				8192.22 (465); *p* < 0.001			
Kaiser-Meyer-Olkin Measure (KMO) (above 0.50)				0.923			
Diagonal element anti-correlation matrix (above 0.50)				0.70–0.98			
Cronbach’s alfa (α) (>0.70)		0.928	0.865	0.818	0.784	0.656	0.822

*h^2^* = extracted communality coefficients; *LD* = structure coefficients; grey; **bold** = items’ saturation.

**Table 4:** Multigroup CFAs of CTL-I according to being or not in a romantic relationship.

	χ^2^	*df*	χ^2^/*df*	RMSEA (CI)	CFI	IFI	SRMR	Comparisons	Δ RMSEA	Δ CFI	Δ SRMR
Configural invariance	1901.57	804	2.365	0.037 (0.035–0.039)	0.924	0.925	0.068	NA	NA	NA	NA
Metric invariance	1953.22	827	2.362	0.037 (0.035–0.039)	0.922	0.923	0.068	Configural vs. metric	0.000	0.002	0.000
Scalar invariance	1959.45	828	2.366	0.037 (0.035–0.039)	0.922	0.922	0.070	Metric vs. Scalar	0.000	0.000	0.002
Error variance invariance	2027.07	843	2.405	0.037 (0.035–0.039)	0.918	0.919	0.070	Scalar vs. error variance	0.000	0.004	0.000

Note. χ^2^ = qui-squared; DF = degrees of freedom; IFI = incremental fit index; CFI = comparative fit index; RMSEA = root mean square error of approximation; CI = confidence interval; SRMS = standard root mean square; Δ RMSEA = change in RMSEA compared with the previous model (expressed in absolute values); Δ CFI = change in CFI compared with the previous model (expressed in absolute values); Δ SRMR = change in SRMR compared with the previous model (expressed in absolute values). All models are significant at *p* < 0.001.

**Table 5:** Correlations, Cronbach’s alpha, McDonald’s omega, composite reliability, average variance extracted (AVE), AVE square roots, mean and standard deviation of the CTL-I.

				Pearson’s Correlations								
	**0**	1	2	3	4	5	6	α	ω	CR	AVE	*Mean* (*SD*)
0. Total	**0.720**							0.888	0.874	0.970	0.519	3.39 (0.35)
1. Interest_Others	0.558 **	**0.722**						0.806	0.802	0.812	0.522	3.84 (0.35)
2. Basic_Trust_Grat	0.858 **	0.519 **	**0.729**					0.922	0.922	0.919	0.531	3.51 (0.56)
3. Common_Ego_Ideal	0.742 **	0.589 **	0.677 **	**0.707**				0.854	0.853	0.837	0.500	3.69 (0.40)
4. Perman_Sexual_Pas	0.452 **	0.069 *	0.274 **	0.211 **	**0.875**			0.830	^a^	0.867	0.766	2.82 (0.92)
5. Loss	0.518 **	0.024	0.173 **	0.101 **	0.237 **	**0.707**		0.659	0.661	0.758	0.500	2.92 (0.58)
6. Mourning	0.427 **	0.009	0.107 **	0.050	0.098 **	0.442 **	**0.810**	0.773	0.776	0.851	0.656	2.85 (0.83)

Note: * *p* < 0.05, ** *p* < 0.001; α = Cronbach’s alpha; ω = McDonald’s omega; CR = composite reliability; AVE = average variance extracted; **bold** (diagonal) = AVE square roots; *SD* = Standard deviation; ^a^ McDonald’s omega cannot be calculated because the number of items is less than 3.

**Table 6:** Variables that contribute to explain CTL-I and its dimensions.

	CTL-I Total			Interest Others			Basic Trust Gratitude			Common Ego Ideal			Permanence Sexual Passion			Loss			Mourning		
	B	EP B	β	B	EP B	β	B	EP B	β	B	EP B	β	B	EP B	β	B	EP B	β	B	EP B	β
Gender	0.068	0.022	0.074	0.129	0.027	0.144				0.097	0.030	0.094	0.156	0.072	0.066						
Age	−0.003	0.001	−0.097	−0.002	0.001	−0.062	−0.006	0.001	−0.133	−0.005	0.001	−0.146	−0.020	0.002	−0.264				0.015	0.002	0.216
Education										0.060	0.022	0.079	−0.110	0.054	−0.063						
Romantic relationship	0.080	0.025	0.099				0.091	0.038	0.072	0.116	0.033	0.128	0.231	0.080	0.110						
SWLLS	0.150	0.007	0.698	0.058	0.006	0.274	0.234	0.010	0.694	0.117	0.009	0.486	0.156	0.021	0.280	0.089	0.011	0.255	0.152	0.015	0.302
R^2^ (R^2^ Adj.)	0.435 (0.432)			0.099 (0.097)			0.458 (0.457)			0.209 (0.205)			0.150 (0.146)			0.065 (0.064)			0.128 (0.127)		
F for change in R^2^	518.30 **			83.94 **			537.44 **			177.40 **			27.91 **			70.50 **			104.47 **		

R^2^ = R squared; R^2^ Adj. = R squared adjusted; B = unstandardized regression coefficients; EP B = unstandardized error of B; β = standardized regression coefficients; ** *p* < 0.001.

**Table 7:** Moderations in the relationship between SWLLS and CTL-I.

Predictor	Moderator	Dependent	*F*(3, 1013)	*p*	β	95% CI	*t*	*p*	Variance %	Moderator Option	β	*p*
SWLLS	Romantic relationship	CTL-I_Total	280.726	<0.001	−0.105	−0.132, −0.078	−7.566	<0.001	67.38	Yes	−0.184	<0.001
SWLLS	Romantic relationship	Interest_Others	39.448	<0.001	−0.094	−0.128, −0.060	−5.420	<0.001	32.34	Yes	−0.085	<0.001
SWLLS	Romantic relationship	Basic_Trust Gratitude	311.862	<0.001	−0.183	−0.225, −0.142	−8.635	<0.001	69.29	Yes	0.299	<0.001
SWLLS	Romantic relationship	Common_Ego Ideal	94.194	<0.001	−0.128	−0.165, −0.092	−6.903	<0.001	46.70	Yes	0.157	<0.001
SWLLS	Romantic relationship	Permanence Sexual Passion	34.735	<0.001	−0.197	−0.288, −0.106	−4.254	<0.001	30.54	Yes	0.253	<0.001
SWLLS	Gender	CTL-I_Total	248.612	<0.001	0.048	0.021, 0.075	3.492	<0.001	65.12	Female	0.146	<0.001
SWLLS	Gender	Basic_Trust Gratitude	272.012	<0.001	0.97	0.056, 0.139	4.619	<0.001	66.79	Female	0.238	<0.001

F = F distribution; *p* = *p*-value; β = standardized beta; CI = confidence interval; *t* = *t*-test.

**Table 8:** Differences between CTL-I and SWLLS means concerning marital status.

Marital Status		CTL-I Total	Interest Others	Basic_Trust Gratitude	Common Ego Ideal	Permanence Sexual Passion	Loss	Mourning	SWLLS
Single	*M*	3.39	3.83	3.53	3.71	2.98	2.91	2.69	4.91
	*SD*	0.34	0.35	0.50	0.39	0.87	0.57	0.83	1.67
Married	*M*	3.43	3.89	3.55	3.70	2.58	2.94	3.15	5.50
	*SD*	0.36	0.28	0.57	0.37	0.94	0.58	0.75	1.41
Separated. divorced. widower	*M*	3.22	3.72	3.17	3.54	2.60	2.97	2.85	4.04
	*SD*	0.43	0.48	0.73	0.51	0.92	0.60	0.86	1.79
*F*		11.40	9.66	18.35	7.03	24.47	0.62	34.46	31.47
*p*		<0.001	<0.001	<0.001	<0.001	<0.001	0.538	<0.001	<0.001
η^2^		0.02	0.02	0.04	0.01	0.05	0.00	0.06	0.06
		(0.01–0.04)	(0.01–0.04)	(0.02–0.06)	(0.00–0.03)	(0.02–0.07)	(0.00–0.01)	(0.04–0.09)	(0.03–0.09)

*M* = mean; *SD* = standard deviation; *F* = variation between sample means; *p* = *p*-value; η^2^ = eta squared size effect.

## Data Availability

The data presented in this study are available on request from the corresponding author.
